# Frontal brain activation changes due to dual-tasking under partial body weight support conditions in older adults with multiple sclerosis

**DOI:** 10.1186/s12984-017-0280-8

**Published:** 2017-06-29

**Authors:** Gioella Chaparro, Julia M. Balto, Brian M. Sandroff, Roee Holtzer, Meltem Izzetoglu, Robert W. Motl, Manuel E. Hernandez

**Affiliations:** 1 Department of Kinesiology and Community Health, University of Illinois at Urbana-Champaign, Champaign, IL USA; 2 Department of Physical Therapy, University of Alabama at Birmingham, Birmingham, AL USA; 3 Department of Neurology, Albert Einstein College of Medicine, Bronx, NY USA; 4 Ferkauf Graduate School of Psychology, Yeshiva University, Bronx, NY USA; 5 School of Biomedical Engineering, Science and Health Systems, Drexel University, Philadelphia, PA USA; 6 Present address: University of Illinois at Urbana-Champaign, 209 Louise Freer Hall, 906 S. Goodwin Avenue, Urbana, IL 61801 USA

**Keywords:** Gait, Attention, Weight-bearing, Functional neuroimaging, Multiple sclerosis

## Abstract

**Background:**

Gait impairments present while dual-tasking in older adults with multiple sclerosis (MS) have been associated with an increased risk of falls. Prior studies have examined prefrontal cortex (PFC) activity using functional near infrared spectroscopy (fNIRS) while dual-tasking in older adults with and without cognitive impairment. While the benefits of partial body weight support (PBWS) on gait have been clearly outlined in the literature, the potential use of PBWS to improve the ability to dual task in older adults with and without MS has not been examined. The aim of this study was to examine the effects of PBWS on the PFC activation while dual-tasking in older adults with and without MS.

**Methods:**

Ten individuals with MS (mean 56.2 ± 5.1 yrs., 8 females) and 12 healthy older adults (HOA) (mean 63.1 ± 4.4 yrs., 9 females) participated in this study. PFC activation (i.e., oxygenated hemoglobin-HbO_2_) was measured using fNIRS. Assessments were done under two treadmill walking conditions: no body weight support (NBWS) and PBWS. Under each condition, participants were asked to walk at a comfortable speed (W) or walk and talk (WT). Linear mixed models were used to test for differences between cohorts, conditions, and tasks.

**Results:**

HbO_2_ levels differed significantly between task (*p* < .001), cohort (*p* < .001), and BWS (*p* = .02). HbO_2_ levels increased under higher cognitive demands (i.e., W vs WT), in individuals with MS, and under different conditions (i.e., NBWS vs PBWS). Post-hoc analysis demonstrated a significant difference between cohorts during the WT and NBWS condition (*p* = .05). When examining the relative change in HbO_2_ levels during each task, a significant interaction between task, BWS, and cohort across time was observed (*p* < 0.01). While HOA increased PFC activation across time, MS exhibited a maintenance of PFC activation patterns during the WT under PBWS condition.

**Conclusions:**

This study establishes the potential impact of PBWS on PFC activation patterns under dual-tasking conditions and sheds light on the ability for PBWS to be used as a therapeutic tool in individuals with neurological conditions to decrease cognitive demands while dual-tasking and thus decrease the risk of falls.

## Background

Walking has traditionally been considered an automatic process controlled by the subcortical and spinal regions [1]. However, recent research based on dual-tasking paradigms (i.e., walking and talking) has established that higher cortical regions are required for walking. For example, when walking and talking, gait rhythmicity and automaticity decrease based on decrements in gait parameters such as reduced gait speed and stride length when compared with walking alone [1–4]. These dual-task-related impairments on gait parameters have been correlated with an increased risk of falls [1, 5–7] and have been well established in healthy older adults (HOA) [4, 8, 9] and in adults with neurological conditions such as multiple sclerosis (MS) [10, 11]. Furthermore, when compared to healthy controls, individuals with MS have greater balance impairments [12–14] which can translate to a greater risk of falls. Falls are the leading cause of injury and hospitalization, which in turn decrease quality of life and are associated with high medical costs [15–17]. Thus, it is essential to examine how to improve dual-task performance and decrease the risk of falls for improving quality of life in HOA and in individuals with MS.

Partial body weight support (PBWS) treadmill training has traditionally been used as an intervention tool for gait rehabilitation purposes in clinical populations such as older adults [18], stroke, [19–22], Parkinson’s disease [23], and spinal cord injury [24]. Many benefits of using PBWS have been found such as improving walking parameters (i.e., increased stride length, walking time, and gait speed and improvements in gait symmetry), decreasing the risk of falls, [23, 25], and promoting confidence [26]. Furthermore, a systematic review established that the use of PBWS leads to improvements in gait speed and distance in individuals with MS [27]. However, it is important to note that these benefits were observed after intervention durations ranging between 4 and 7 weeks. In individuals with and without MS, the inability to effectively divide attentional resources for both the motor and cognitive tasks can lead to the decreased walking speeds observed while dual-tasking [1, 28–30]. Theoretically, because of the harness support under PBWS, the postural demands of walking are reduced which can lead to reductions in the amount of attentional demands required for dual-tasking. However, the effects of a single session of PBWS on dual-tasking in HOA and MS, crucial for establishing a baseline understanding of the changes in attentional demands that can occur, has not been examined.

Using neuroimaging techniques such as functional near-infrared spectroscopy (fNIRS), studies have established that the prefrontal cortex (PFC), in particular, is responsible for overseeing and coordinating attentional resources while dual-tasking [31–35]. With cognitive function declines, there are age-related activation changes in the PFC that are associated with executive dysfunction and fall risk [36–39]. Furthermore, research has examined the effects of dual-tasking on the PFC activation patterns in older adults. Higher concentrations of oxygenated hemoglobin (HbO_2_) (i.e., increased PFC activation) have been reported during the dual-task or more difficult cognitive task when compared to the single task or easier cognitive task in older adults with Mild Cognitive Impairment [40] and in HOA when compared to younger healthy counterparts [41, 42]. Furthermore, in a study published by Hernandez et al. (2016), individuals with MS exhibited greater oxygenation levels while they took part in a dual task during over ground walking when compared to healthy controls. However, the effect of dual-tasking on PFC oxygenation levels in older adults with and without MS while walking on a self-paced treadmill under PBWS, important for providing insight into potential mechanisms underlying dual-tasking improvements under PBWS, has not been examined. Thus, a study examining the impact of a single session of PBWS on attentional demands provides future researchers and clinicians with insight crucial for furthering the development of interventions using PBWS treadmill training in persons with MS.

The present study aims to 1) better understand how PFC activation patterns differ between MS and HOA in a single session of PBWS and 2) to validate prior findings of PFC activation changes in MS and HOA on self-paced treadmill walking using fNIRS. To our knowledge, this study is the first to examine changes in PFC activation levels due to PBWS using fNIRS. In this study, we hypothesized that changes in PFC activation levels would be detected in a single session of PBWS, when compared to no body weight support (NBWS), in HOA and MS, particularly during dual-task conditions. These findings would provide clinicians and rehabilitation specialists insight on the attentional demands required for dual-tasking in older adults with MS and on the impact of the one-time use of PBWS on dual-tasking. As benefits that are observed over a one-time session of dual-tasking under PBWS would be expected to be further enhanced after an intervention program, this study provides potential measures for future intervention programs using dual-task training under PBWS conditions.

## Methods

### Participants

Ten community dwelling older adults with MS (mean 56.2 ± 5.1 yrs.; range: 49–62 yrs., 8 females; higher education level: 3.9 ± 2.9 yrs) and 12 HOA (mean 63.1 ± 4.4 yrs.; range: 53–68 yrs., 9 females; higher education level: 4.9 ± 3.1 yrs) were recruited from the surrounding community to participate in the study. Subjects were included in the study if they had no lower limb injury in the past 6 months and were medically stable, had normal or corrected to normal vision, and were over 45 years of age. Subjects were excluded if they had any cognitive dysfunction, any neurological disorders other than MS, or a relapse within the last 30 days for the MS group. All participants with MS had mild to moderate disability, as evaluated by the Kurtzke Expanded Disability Status Scale (EDSS) [43]. Overall eligibility to participate in the study was established through a health screening and a score above 18 on the Telephone Interview for Cognitive Status (TICS-M) [44]. All participants were informed about the study and signed informed consent forms approved by a university-based institutional review board before data collection.

### Procedure

**Fig. 1:**
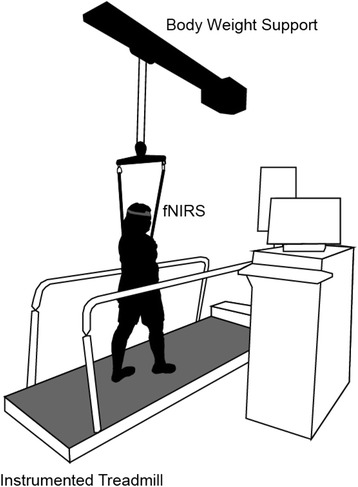
Experimental setup

A three-meter self-paced treadmill (C-Mill, Motekforce Link, Culemborg, The Netherlands) was utilized for all walking conditions. For safety purposes, all participants wore a harness during all walking conditions (see Fig. 1). Spatiotemporal gait characteristics such as step length, stride length (SL), stride time (ST), and stride frequency (SF) were recorded by the CueFors 2 software and used to calculate average treadmill gait speed (GS) (Motekforce Link, Culemborg, The Netherlands). Because of the unique self-paced feature of the C-Mill, participants were given instruction on the ability of the treadmill to adjust to their comfortable speed and began with an acclimation trial to adjust to walking on the treadmill and the body weight support system, before testing began.

**Fig. 2:**
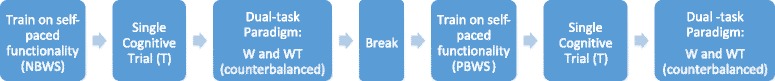
Protocol diagram

Participants performed the dual-task paradigm while wearing an fNIRS headband under two different conditions: one with NBWS and the other with PBWS. The amount of body weight support provided for each participant during the PBWS conditions was defined as 30% of their weight [45]. All participants experienced the dual-task paradigm under NBWS condition first, followed by the PBWS condition (Fig. 2). The dual-task paradigm consisted of one trial of each of the following counterbalanced conditions: walking (W) and walking and talking (WT). For the WT condition, participants were asked to recite alternating letters of the alphabet [9]. To compare this dual-task condition to a cognitive single task condition, participants were asked to recite alternate letters of the alphabet while standing still (Talking). For each cognitive trial, participants were given different and randomized letters to start the alphabet recitation. A research assistant recorded the amount of utterances to calculate the utterance rate (number of correct utterances divided by the trial time). Each walking trial consisted of a 30 s warmup period to reach a comfortable self-paced speed, followed by a 30 s period used for testing, and a 15 s period to decelerate. There was a 10 s baseline before each condition, which consisted of the participant standing still and counting silently in their head [46]. Participants were measured for weight and height and completed further demographic questionnaires. Weight and height were later used to calculate body mass index (%- BMI).

### fNIRS assessment

The fNIR sensor headband (fNIR Imager 1000, fNIR Devices LLC, Potomac, MD) was used to collect hemodynamic activity in the PFC. The headband consisted of 10 photodetectors and 4 LED light sources with a 2.5 cm source-detector separation distance that provided coverage of the forehead with 16 optodes and a 2 Hz sampling rate. The center of the headband sensor was placed on the central point of the forehead above the nasion (Fpz); in accordance with the 10/20 system used in electroencephalography. HbO_2_ levels were used for this study because of its reliability established for being a direct reflection of cortical activation and the most reliable for measuring gait-related changes in cerebral oxygenation [40, 47–49]. The task-related changes were measured by averaging the HbO_2_ during the walking and comparing it to baseline values under each condition [42]. A software package (E-prime, Psychology Software Tools, Inc., U.S.A.) was used to synchronize the start and end of each walking trial and cognitive task with the fNIRS system [50].

### Cognition and physical function

The following measures were used in this study to account for potential confounding factors. The Repeated Battery for the Assessment of Neuropsychological Status (RBANS) was implemented to assess cognitive function [51] and the Short Physical Performance Battery (SPPB) to assess physical function [52]. For these tests, higher scores indicated higher cognitive and physical function, respectively. To capture an additional measure of physical function, over ground gait speed (m/s) was measured. Participants were asked to walk 3 loops at a comfortable pace on a walkway until instructed to stop. As described in prior studies [46, 53, 54], a research assistant followed closely behind each participant during the 3 loops with a measuring wheel (Stanley MW50, New Briton, CT) to accurately record the actual distance traveled (m). Over ground gait speed was then calculated by dividing the actual distance traveled by the time elapsed from the start to the stop of three consecutive loops. To further understand the effects of dual-tasking under the different BWS conditions, the dual task cost (DTC) on each of the gait parameters was calculated. The DTC was calculated as ((W-WT/W) × 100). A positive value for GS, SL, and SF and a negative value for ST indicate a decrement in gait parameters while dual-tasking.

### Data analysis

The fNIR data were processed by an offsite researcher who was uninvolved in data collection and other experimental procedures (i.e., blinded fNIRS data analysis). The data were acquired through the COBI Studio software and processed and analyzed using MATLAB R2014a (The Mathworks, Inc.). The raw data were visually inspected for excessive noise, saturation or dark current conditions. To remove the physiological effects and any additional noise, the raw data were filtered using a low-pass filter with a cut-off frequency at 0.14 Hz [55]. HbO_2_ levels were calculated by using the modified Beer-Lambert law for each of the 16 channels [56]. Spatiotemporal data from the CueFors 2 software were exported to MATLAB R2014a (The Mathworks, Inc.). Custom MATLAB scripts were used for processing data and exporting to R. The gait parameters that were analyzed in this study included GS, SL, ST, and SF. R, version 3.1.1 [57] was used to run the statistical analysis with the significance level set at .05. Independent t-tests were run to analyze cohort differences in demographic and behavioral data. A χ^2^ test was used to examine gender differences between the groups. A linear mixed model was used to examine differences between cohort (MS and HOA), task (W and WT) and BWS (NBWS and PBWS) in spatiotemporal gait parameters and HbO_2_ activation levels during the 30 s testing period. The linear mixed models controlled for age and were examined using the *lme4* package in R [58]. Random intercepts were included: by-individuals, by-BWS, and by-task across optodes. A secondary exploratory analysis was carried out to examine potential confounding factors related to increases in HbO_2,_ such as age, and physical and cognitive function. RBANS, over ground gait speed, and SPPB scores were divided into tertiles and were considered potential predictors for changes in HbO_2_ levels in univariate linear mixed models. To examine any potential correlations between the gait parameters and HbO_2_ levels, Pearson correlations were run. A linear mixed model was used to examine the effect of DTC, cohort, and BWS on each of the gait parameters. Lastly, we examined the relative change in HbO_2_ levels across each task, by examining the change from the first to the last 10 s throughout the course of each 30 s trial (i.e., relative change in HbO_2_ levels = average HbO_2_ value from the last 10 s minus the average from the first 10 s). A linear mixed model was used to examine the effect of task, cohort, and BWS on HbO_2_ levels across time. Data was visually inspected and the assumptions for the linear mixed model test were met. To control for multiple comparisons, post-hoc *t*-tests were carried out using Hochberg’s step-up method.

## Results

Overall, the MS cohort was younger (56.2 vs 63.1 years, *p* < .01) and had lower physical function scores on the SPPB (10.2 vs. 11.6, *p* = .04) when compared to HOA. There were no significant group differences in any of the other demographic measures (Table 1). Behavioral data including treadmill gait speed and utterance rate are provided in Table 2 for each task and condition. Based on a linear mixed-effects model, stride length exhibited a significant 7% decrease from NBWS to PBWS conditions (*p* = .01). Additionally, there was an 11% increase in stride length observed in MS when compared to HOA, though it was not statistically significant (*p* = .24). There was a significant two-way interaction in the stride time between BWS conditions and cohort (*p* = .04). MS exhibited slower stride times during the NBWS condition when compared to HOA and PBWS. There were no statistically significant differences observed in gait speed or stride frequency. Results of the DTC on each of the gait parameters are demonstrated on Table 3. Significant differences across cohorts are consistent with previous literature found where the individuals with MS demonstrated a greater DTC (i.e., slower GS and shorter SL) when compared to HOA (*p* < .05) [28]. There was a significant effect on BWS for the DTC on GS, ST, and SF (*p* < .05); there was a higher DTC during PBWS conditions when compared to NBWS conditions. Lastly, there was a significant two-way interaction between BWS and cohort for the DTC on SL where the MS demonstrated the greatest DTC during PBWS conditions when compared to HOA and NBWS.

**Table 1:** Demographics and characteristics of participants

Characteristic	MS (*n* = 10)	HOA (*n* = 12)	*p*-value
Age (yr)	56.2 (5.1)	63.1 (4.4)	0.004**
Sex (# of females)	8	9	1.00
Height (m)	1.69 (0.09)	1.7 (0.09)	0.85
Weight (kg)	70.4 (9.6)	77.6 (17.99)	0.4
BMI (%)	24.72 (3.83)	26.9 (6.23)	0.34
Higher education (yr)	3.85 (2.9)	4.9 (3.1)	0.41
SPPB (0–12)	10.2 (1.8)	11.6 (.7)	0.04*
RBANS (40–160)	98.1 (13.43)	105.6 (11.3)	0.18
Overground gait speed (m/s)	.87 (.22)	.94 (.12)	0.44
EDSS (0–10)	3.7 (1.6)		

*Abbreviations MS* Multiple Sclerosis, *HOA* Healthy older adults, *BMI* Body mass index, *SPPB* Short physical performance battery *RBANS* Repeated Battery for the Assessment of Neuropsychological Status, *EDSS* Expanded Disability Status Scale

Mean (SD) and results from independent *t* test. *significant difference at *p* < .05. **significant difference at *p* < .005

**Table 2:** Summary of primary motor and cognitive performance measures

Treadmill Gait Speed (m/s)				
	W	W-PBWS	WT	WT-PBWS
HOA	1.00 (.23)	1.06 (.29)	0.94 (.29)	0.93(.20)
MS	1.06 (.34)	1.22 (.34)	1.18 (.33)	1.17 (.36)
*p*-value	0.62	0.27	0.10	0.08
Utterance Rate (# correct utterances/time [s])				
	Talking	WT	WT-PBWS	
HOA	.62 (.19)	.50 (.18)	.49 (.18)	
MS	.65 (.17)	.55 (.14)	.54 (.12)	
*p*-value	0.66	0.48	0.42	

*Abbreviations W* Walking, *W-PBWS* Walking under partial body weight support, *WT* Walking and talking, *WT-PBWS* Walking and talking under partial body weight support, *HOA* Healthy older adults, *MS* Multiple Sclerosis

Mean (SD) values of treadmill gait speed and utterance rate and results from independent t test grouped by condition

**Table 3:** Summary of the dual task cost effects on the gait parameters

	GS *, ǂ		SL ǂ, #		ST*		SF*	
	NBWS	PBWS	NBWS	PBWS	NBWS	PBWS	NBWS	PBWS
HOA	5.15 (22.12)	9.89 (16.07)	4.65 (16.09)	3.57 (12.99)	−2.10 (8.90)	−8 (6.04)	1.46 (8.73)	7.17 (4.97)
MS	−15.35 (23.09)	3.89 (9.26)	−14.15 (15.26)	−1.35 (5.76)	−.22 (8.39)	−6.01 (7.88)	−.45 (8.94)	5.29 (6.25)

*Abbreviations GS* Gait speed, *SL* Stride length, *ST* Stride time, *SF* Stride frequency, *MS* Multiple Sclerosis, *HOA* Healthy older adults, *NBWS* No body weight support, *PBWS* Partial body weight support

Mean (SD) dual task costs of gait parameters and results from linear mixed-effects model. * significant difference between BWS at *p* < .05. ǂ significant difference between cohorts at *p* < .05. # significant interaction between BWS and cohort at *p* < .05

**Fig. 3:**
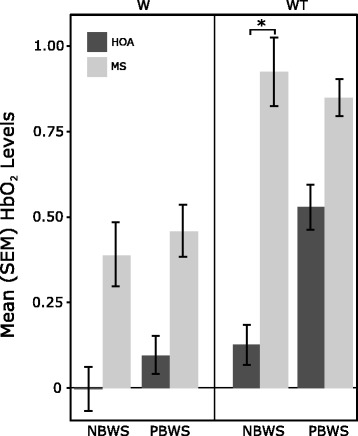
Oxygenated hemoglobin (HbO_2_) levels during walking (W) and walking while talking (WT) tasks in no body weight support (NBWS) and partial body weight support (PBWS) conditions in older adults with multiple sclerosis (MS) and healthy older adults (HOA). *Indicates a significant cohort effect (*p*-value < .05) in Hochberg’s post-hoc tests

HbO_2_ levels differed significantly between task (*p* < .001), cohort (*p* < .001), and BWS conditions (*p* < .01). In general, HbO_2_ levels were significantly higher under dual-task conditions (i.e., WT vs W), for cohort (i.e., MS vs HOA), and for BWS (i.e., PBWS vs NBWS) conditions. There were significant two-way interactions between: 1) task and cohort (*p* = .03), where MS exhibited larger increases in HbO_2_ levels as task difficulty increased (i.e., during WT) in comparison to HOA; and 2) cohort and BWS (*p* < .01), where HOA exhibited consistent increases in HbO_2_ levels as PBWS was provided when compared to MS. Lastly, a significant three-way interaction was found between task, cohort, and BWS (*p* < .01). As seen in Fig. 3, individuals with MS exhibited significantly higher activation levels during NBWS and WT conditions when compared to HOA. Post hoc results indicated a significant difference (*p* = .05) between the cohorts under the WT-NBWS condition only, which may help explain the significant interaction observed between task and cohort.

To rule out factors that have been found to be correlated with increases in HbO_2_ activation levels, a linear mixed model was run with RBANS, over ground gait speed, and SPPB as potential predictors for changes in HbO_2_ levels. There were no significant effects between the potential predictors for increases in activation levels. These tests allowed us to conclude that the increases in activation levels observed within cohorts were not simply due to differences in age, physical function (over ground gait speed and SPPB), or cognitive function (RBANS). There were no significant correlations between any of the gait parameters and the HbO_2_ levels.

**Fig. 4:**
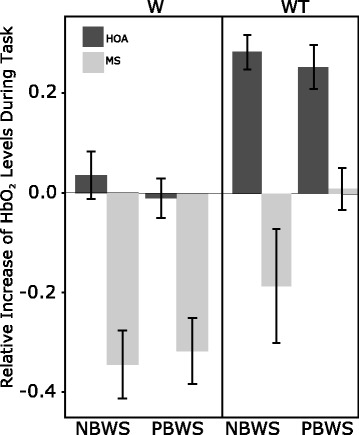
Comparison between relative change in oxygenated hemoglobin (HbO_2_) levels during walking (W) and walking while talking (WT) tasks in no body weight support (NBWS) and partial body weight support (PBWS) conditions in older adults with multiple sclerosis (MS) and healthy older adults (HOA). The relative change in HbO_2_ levels was examined using the change from the first to the last 10 s throughout the course of each 30 s trial (i.e., relative change in HbO_2_ levels = average HbO_2_ value from the last 10 s minus the average from the first 10 s). A significant interaction between task, BWS, and cohort across time (*p*-value < .001) was exhibited from the Hochberg’s post-hoc tests

The relative change in HbO_2_ levels from the first to the last 10 s of each trial was used to observe any potential plateaus or increases in activation levels across time in each condition. Higher activation levels during the last 10 s compared to the first 10 s of a trial indicated an upregulation in PFC activation levels across the trial. As observed in Fig. 4, HOA exhibited relative increases across W-NBWS, WT-NBWS, and WT-PBWS trials. In contrast, lower activation levels during the last 10 s when compared to the first 10 s of a trial indicated a downregulation in PFC activation levels across the trial. In Fig. 4, this behavior is exhibited in the MS group during W-NBWS, W-PBWS, and WT-NBWS. Lastly, similar levels of activation during the last 10 s when compared to the first 10 s indicate the maintenance of PFC activation levels across the trial. This can be observed in Fig. 4 in HOA during W-PBWS and in MS during the WT-PBWS trials. Furthermore, these findings exhibited a significant interaction between task, BWS, and cohort across time (*p* < .001). Surprisingly, as task difficulty increased and PBWS was provided, HOA increased PFC activation across time while MS maintained PFC activation patterns as shown in Fig. 4.

## Discussion and conclusions

This study examined PFC activation patterns while dual-tasking under the use of PBWS in older adults with and without neurological conditions. Changes in HbO_2_ were measured using fNIRS during a dual-task paradigm and with different BWS conditions. These activation measures were further examined by making comparisons between the single and dual tasks and NBWS and PBWS conditions. The current results demonstrated: 1) activation levels were significantly higher during dual task conditions when compared to single task, 2) MS exhibited significantly higher activation than HOA across all tasks and conditions, 3) in HOA only, activation levels significantly increased as task difficulty increased under PBWS when compared to NBWS conditions, and 4) HOA increased PFC activation during the course of WT conditions while MS were unable to maintain their PFC activation levels across WT conditions, unless provided with PBWS. Taken together, these findings are consistent with previous literature examining PFC activation patterns during dual-tasking and shed light on the potential benefits that individuals with MS can gain from a single session of PBWS under dual-task conditions.

The use of PBWS was implemented in this study to examine if decreasing the physical load on individuals would alter the attentional demands required while dual-tasking and thus lead to changes in PFC activation levels. Overall, there was a significant three-way interaction between task, cohort, and BWS (Fig. 3) where MS exhibited higher PFC activation levels during NBWS and WT conditions when compared to HOA. Similar results have been observed in MS while dual-tasking during over ground walking [46]. Fig. 4 illustrates the relative change in PFC activation across the trial (i.e., comparing the last ten seconds to the first ten seconds of the trial), and allows us to gain a better understanding of the PFC activation during the trials across the cohorts (see Fig. 4). The down regulation observed in MS during the W trials and WT-NBWS can be indicative of an inability to activate enough PFC activation patterns to maintain task performance. Fig. 3 illustrated the lower mean HbO_2_ levels in MS during these trials and thus helps iterate the behavior exhibited in Fig. 4. The individuals with MS, a group that showed significantly lower physical function scores (see SPPB scores on Table 1) when compared to their healthy counterparts, demonstrated the least amount of change and thus demonstrated an ability to maintain PFC activation only during WT-PBWS, when compared to all other trials. Thus, this behavior indicates that when asked to dual-task, persons with MS are able to recruit and maintain PFC activation levels so as to maintain similar task performance when they are introduced to a tool that assisted them with their walking and decreased the physical load on them. These findings suggest that the use of PBWS may cause a therapeutic effect, which allows individuals with limitations in physical function to maintain their PFC activation levels. The observed maintenance of PFC activation levels under WT-PBWS conditions furthers the motivation for implementing PBWS in individuals with physical impairments. Meanwhile, during WT-PBWS, HOA demonstrated relative increases in PFC activation across the trial. In a healthy sample, perhaps implementing a new tool such as that as PBWS can cause a detrimental effect and hinder their automatic walking processes. Thus, leading to decreases in automaticity which can be reflected by the need to recruit more resources and increase activation throughout the WT-PBWS trial, as was seen in HOA in Fig. 4.

Overall, higher PFC activation levels were observed during a single session of PBWS, relative to NBWS conditions. Possible explanations for increases in PFC activation patterns under PBWS could be due to the novel set-up and function of the treadmill. Along with being supported by the harness at 30% of the weight, the self-paced functionality is something the participants had never been exposed to and required learning. Though training was provided beforehand, it is possible that there was insufficient training to capture natural automatic behavior. Studies have established differences in activation patterns when it comes to automaticity and learning a new task. Newly learned tasks have been found to be correlated with increases in activation while overlearned task performance has no activation [59–61]. A task is referred to being automatic when it is overlearned and is not influenced by the performance of a secondary task [62], or does not cause a significant increase in PFC activation levels [63]. Furthermore, the significant differences observed in the DTC of GS, ST, and SF (i.e.: participants walked slower, had a slower stride time, and lower stride frequency) during PBWS conditions when compared to NBWS helps further explain this behavior. In this study, the novel task of walking on a self-paced treadmill as well as using PBWS was a new condition to learn and was not automatic which may help explain the increases in PFC activation levels across all trials in both cohorts and the higher DTC under PBWS. Surprisingly, HOA exhibited higher PFC activation levels during WT-PBWS conditions, which could be partly explained by a decreased automaticity in the control of self-paced treadmill walking.

The increases in PFC activation observed when dual-tasking (i.e., from W to WT) are consistent with previous studies examining older adults [42, 64, 65], individuals with Mild Cognitive Impairment [40], and stroke [66]. Due to the higher cognitive loads required to dual-task, HOA exhibit increases in activation patterns when compared to healthy younger adults [67]. This behavior in PFC activation has been explained by two main theories. The Compensation-Related Utilization of Neural Circuits Hypothesis (CRUNCH) explains that due to less efficient neural processing, older adults need to compensate and recruit more areas of the brain than younger adults to maintain task performance at high levels of cognitive demands, which leads to increased brain activation [67]. The neural inefficiency model explains the need for older adults to activate the same areas of the brain to a higher degree than younger adults to maintain a similar level of cognitive performance [68]. Lastly, these findings were consistent with studies examining fMRI where individuals with MS exhibited greater cortical activation (i.e., BOLD) when compared to healthy controls [69] and recruited more areas [70, 71] for a given task. Based upon this knowledge, it was hypothesized that persons with MS would need to recruit more areas of the brain and activate more to be able to dual-task and thus demonstrate higher PFC activation levels when compared to HOA.

In this study, MS participants exhibited higher activation patterns in all conditions (i.e., task and BWS) when compared to HOA. Due to the attentional problems associated with MS [72], the CRUNCH and neural inefficiency models would help explain why persons with MS recruit more areas of the brain in order to perform the same task as HOA. It is important to note though, that in this study there were no significant differences observed in cognitive status as measured by the RBANS. Thus, in this study, the increase in PFC activation observed in MS when compared to HOA can alternatively be explained by the decreased physical function observed. Due to the significantly lower physical function scores (i.e., SPPB), it can be concluded that MS demonstrated slower walking speeds, chair stands, or lower balance scores when compared to HOA and thus can indicate the need to recruit or activate more of the PFC to maintain dual-task performance. Furthermore, the significant difference in the DTC of GS and SL across cohorts demonstrating that MS had greater decrements in their gait (i.e., walked slower and had smaller stride lengths) while dual-tasking when compared to HOA can further explain this behavior. Increases in activation observed in older adults have been positively correlated to faster walking speeds [73]. The non-significant correlations found between treadmill gait speed and PFC activation levels eliminate this potential explanation for increases in PFC activation observed in MS. In addition, other potential predictors for increases in HbO_2_ such as age, over ground gait speed, and cognitive function (RBANS) were not found to have a significant effect.

This study is not without limitations. The small sample size recruited for this study did not allow us to make general recommendations representing the MS community. Due to the large amount of statistical analyses that were done within this study, there is the possibility that Type I errors exist. The non-homogenous sample (i.e., significant differences in age) collected may have led to the differences observed in activation levels between the cohorts. Future studies should focus on examining a larger sample size with similar ages to allow examination of PFC activation related to neurological disability. It is important to recognize that the control of locomotion is dependent on numerous brain regions and not just the PFC [74–76]. Thus, only examining the PFC in this study is a limitation. In this study, the fact that the tasks (i.e., W and WT) were pseudorandomized for each participant but the BWS conditions were not (i.e., NBWS conditions were done before the PBWS) would not help explain the differences observed in oxygenation levels across these conditions. As seen in Fig. 3, only the HOA increased activation during the WT trials from NBWS to PBWS. Possible explanations for not observing the hypothesized effects under PBWS may have been due to the insufficient amount of training the participants received with PBWS and on the ability to self-pace walk. Future studies should focus on more training time with novel functions such as self-paced walking and the use of PBWS to allow individuals to feel more comfortable under this novel function and thus exhibit more naturalistic walking behaviors and impact PFC activation patterns differently. Lastly, because this was a cross-sectional design study, it is important to note that the amount of time spent under PBWS conditions might have been insufficient to capture any significant positive effects on PFC due to the use of PBWS. Future studies should perhaps focus on an intervention using PBWS to examine the benefits associated with PBWS and dual-tasking. Due to the nature of the aims of this study, analysis on treadmill gait speed across the different conditions were not examined. In addition, future studies examining the potential benefits of PBWS should consider counterbalanced designs or fixed-sequence designs (e.g., NBWS, PBWS, NBWS) to examine learning or familiarization confounds in greater depth.

This was the first study to date to examine the effect of PBWS on PFC activation patterns while dual-tasking in older adults with and without MS. Findings exhibited that while HOA increased PFC activation when performing under PBWS vs NBWS, individuals with MS were able to maintain PFC activation levels. These findings may suggest that the use of PBWS can serve as a therapeutic tool for individuals with neurological conditions. PBWS conditions allowed for the maintenance of PFC activation patterns in MS which does not lead to an overexertion of physical demands and thus might translate to reducing the risk of falls while dual-tasking. Findings examining the PFC activation patterns while dual-tasking in individuals with neurological conditions exhibited that MS had higher PFC activation patterns across tasks and BWS conditions when compared to the HOA. These findings suggest that due to the physical impairments associated with MS, increases in PFC activation are observed and shine light on the importance for clinicians to implement more physically-based rehabilitation in individuals with MS. Having lower physical function can lead to decreases in PFC activation, which may ultimately lead to a decrease in the risk of falls while dual-tasking.

## Abbreviations

BMI: Body mass index
CRUNCH: Compensation-related utilization of neural network hypothesis
EDSS: Expanded Disability Status Scale
fNIRS: functional near infrared spectroscopy
HbO_2_: Oxygenated hemoglobin
HOA: Healthy older adults
MS: Multiple Sclerosis
NBWS: No partial body weight support
PBWS: Partial body weight support
PFC: Prefrontal cortex
RBANS: Repeated Battery for the Assessment of Neuropsychological Status
SPPB: Short Physical Performance Battery
TICS-M: Telephone Interview for Cognitive Status
W: Walking
WT: Walking and talking
WT-NBWS: Walking under no body weight support
WT-PBWS: Walking and talking under partial body weight support

## References

[1] Yogev N, Giladi C, Peretz S, Springer ES, Hausdorff JM (2005). Dual tasking, gait rhythmicity, and Parkinson’s disease: which aspects of gait are attention demanding?. Eur J Neurosci.

[2] Woollacott M, Shumway-Cook A (2002). Attention and the control of posture and gait: a review of an emerging area of research. Gait Posture.

[3] Brauer SG, Woollacott M, Shumway-Cook A (2002). The influence of a concurrent cognitive task on the compensatory stepping response to a perturbation in balance-impaired and healthy elders. Gait Posture.

[4] Dubost V, Kressig RW, Gonthier R (2006). Relationships between dual-task related changes in stride time variability in healthy older adults. Hum Mov Sci.

[5] Yogev-Seligmann G, Hausdorff JM, Giladi N (2008). The role of executive function and attention in gait. Mov Disord.

[6] Hausdorff JM, Rios DA, Edelberg HK (2001). Gait variability and fall risk in community-living older adults: A 1-year prospective study. Arch Phys Med Rehabil.

[7] Wajda DA, Motl RW, Sosnoff JJ (2013). Dual task cost of walking is related to fall risk in persons with multiple sclerosis. J Neurol Sci.

[8] Beauchet O, Dubost V, Gonthier R, Kressig RW (2005). Dual-task related changes in transitionally frail older adults: The type of the walking-associated cognitive task matters. Gerontology.

[9] Verghese J, Buschke H, Viola L (2002). Validity of divided attention tasks in predicting falls in older individuals: A preliminary study. J Am Geriatr Soc.

[10] Sosnoff JJ, Socie MJ, Sandroff BM (2014). Mobility and cognitive correlates of dual task cost of walking in persons with multiple sclerosis. Disabil Rehabil.

[11] Motl RW, Sosnoff JJ, Dlugonski D, Pilutti LA, Sandroff BM (2014). Walking and cognition, but not symptoms, correlate with dual task cost of walking in multiple sclerosis. Gait Posture.

[12] Soyuer F, Mirza M, Erkorkmaz U (2006). Balance performance in three forms of multiple sclerosis. Neurol Res.

[13] Spain RI, St George RJ, Salarian A (2012). Body-worn motion sensors detect balance and gait deficits in people with multiple sclerosis who have normal walking speed. Gait Posture.

[14] Cl M, Phillips BA, Kilpatrick TJ (2006). Gait and balance impairment in early multiple sclerosis in the absence of clinical disability. Mult Scler.

[15] Kannus P, Sievanen H, Palvanen M, Jarvinen T, Parkkari J (2005). Prevention of falls and consequent injuries in elderly people. Lancet.

[16] Rubenstein LZ (2006). Falls in older people: epidemiology, risk factors and strategies for prevention. Age Ageing.

[17] Holtzer R, Friedman R, Lipton RB (2007). The relationship between specific cognitive functions and falls in aging. Neuropsychology.

[18] Peterson MJ, Williams N, Caves K, Morey MC. A pilot study of partial unweighted treadmill training in mobility-impaired older adults. BioMed Res Int. 2014; 321048. 10.1155/2014/321048 PMC3950400 24701568

[19] Park B, Kim M, Lee L (2015). Effects of conventional overground gait training and a gait trainer with partial body weight support on spatiotemporal gait parameters of patients after stroke. J Phys Ther Sci.

[20] Burnfield JM, Buster TW, Goldman AJ, Corbridge LM, Harper-Hanigan K (2016). Partial body weight support treadmill training speed influences paretic and non-paretic leg muscle stride characteristics, and ratings of perceived exertion during acute stroke rehabilitation. Hum Mov Sci.

[21] Visintin M, Barbeau H, Korner-Bitensky N (1998). A new approach to retrain gait in stroke patients through body weight support and treadmill stimulation. Stroke.

[22] Sousa CO, Barela JA, Prado-Medeiros CL, Salvini TF, Barela AM (2011). Gait training with partial body weight support during overground walking for individuals with chronic stroke: a pilot study. J NeuroEng Rehabil.

[23] Miyai I, Fujimoto Y, Ueda Y (2000). Treadmill training with body weight support: its effect on parkinson's disease. Arch Phys Med Rehabil.

[24] Field-Fote EC (2001). Combined use of body weight support, functional electric stimulation, and treadmill training to improve walking ability in individuals with chronic incomplete spinal cord injury. Arch Phys Med Rehabil.

[25] Ribeiro T, Britto H, Oliveira D, Silva E, Galvao E, Lindquist A (2013). Effects of treadmill training with partial body weight support and the proprioceptive neuromuscular facilitation methods of hemiparetic gait: a randomized controlled study. Eur J Phys Rehabil Med.

[26] Hesse S, Helm B, Krajnik J (1997). Treadmill training with partial body weight support: influence of body weight release on the gait of hemiparetic patients. J Neurol Rehabil.

[27] Swinnen E, Beckwee D, Pinte D, Meeusen R, Baeyens J, Kerckhofs E. Treadmill training in multiple sclerosis: Can body weight support or robot assistance provide added value? A systematic review. Mult Scler Int. 2012; 240274. 10.1155/2012/240274 PMC3369491 22701177

[28] Learmonth YC, Sandroff BM, Pilutti LA, Klaren RE, Ensari I, Riskin BJ, Holtzer R, Motl RW (2014). Cognitive motor interference during walking in multiple sclerosis using an alternate-letter alphabet task. Arch Phys Med Rehabil.

[29] Sosnoff JJ, Boes MK, Sandroff BM, Socie MJ, Pula JH, Motl RW (2011). Walking and thinking in persons with multiple sclerosis who vary in disability. Arch Phys Med Rehabil.

[30] Hall CD, Echt KV, Wolf SL, Rogers WA (2011). Cognitive and motor mechanisms underlying older adults’ ability to divide attention while walking. Phys Ther.

[31] Holtzer R, Stern Y, Rakitin BC (2004). Age-related difference in executive control of working memory. Mem Cogn.

[32] Hausdorff JM, Schweiger A, Herman T, Yogev-Seligmann G, Giladi N (2008). Dual-task decrements in gait: contributing factors among healthy older adults. J Gerontol A Biol Sci Med Sci.

[33] Stelzel C, Brandt SA, Schubert T (2009). Neural mechanisms of concurrent stimulus processing in dual tasks. NeuroImage.

[34] Herman T, Mirelman A, Giladi N, Schweiger A, Hausdorff JM (2010). Executive control deficits as a prodrome to falls in healthy older adults: A prospective study linking thinking, walking, and falling. J Gerontol A Biol Sci Med Sci.

[35] Turner GR, Spreng RN (2012). Executive functions and neurocognitive aging: dissociable patterns of brain activity. Neurobiol Aging.

[36] Smith E, Jonides J (1999). Storage and executive processes in the frontal lobes. Science.

[37] O’Sullivan M, Jones D, Summers P, Morris R, Williams S, Markus H (2000). Evidence for cortical “disconnection” as a mechanism of age-related cognitive decline. Neurology.

[38] Soumare A, Elbaz A, Zhu A (2009). White matter lesions volume and motor performances in the elderly. Ann Neurol.

[39] Anstey KJ, Wood J, Kerr G, Caldwell H, Lord SR (2009). Different cognitive profiles for single compared with recurrent fallers without dementia. Neuropsychology.

[40] Doi T, Makizako H, Shimada H (2013). Brain activation during dual-task walking and executive function among older adults with mild cognitive impairment: a fNIRS study. Aging Clin Exp Res.

[41] Langenecker SA, Nielson KA, Rao SM (2004). fMRI of healthy older adults during Stroop interference. NeuroImage.

[42] Ohsugi H, Ohgi S, Shigemori K, Schneider EB (2013). Differences in dual-task performance and prefrontal cortex activation between younger and older adults. BMC Neurosci.

[43] Kurtzke JF (1983). Rating neurologic impairment in multiple sclerosis: An expanded disability status scale (EDSS). Neurology.

[44] Welsh KA, Breitner JCS, Magruder-Habib KM (1993). Detection of dementia in the elderly using telephone screening of cognitive status. Neuropsychiatr Neuropsychol Behav Neurol.

[45] Lindquist AR, Prado CL, Barros RM, Mattioli R, da Costa PH, Salvini TF (2007). Gait training combining partial body-weight support, a treadmill, and functional electrical stimulation: effects on poststroke gait. Phys Ther.

[46] Hernandez ME, Holtzer R, Chaparro G, Jean K, Balto JM, Sandroff BM, Izzetoglu M, Motl RW. Brain activation changes during locomotion in middle-aged to older adults with multiple sclerosis. J Neurol Sci. 2016;370:277-283. 10.1016/j.jns.2016.10.002 27772776

[47] Miyai I, Tanabe HC, Sase I (2001). Cortical mapping of gait in humans: a near-infrared spectroscopic topography study. NeuroImage.

[48] Strangman G, Granceschini MA, Boas DA (2003). Factors affecting the accuracy of near-infrared spectroscopy concentration calculations for focal changes in oxygenation parameters. NeuroImage.

[49] Vermeij A, van Beek AH, Olde Rikkert MG, Claassen JA, Kessels RP (2012). Effects of aging on cerebral oxygenation during working-memory performance: a functional near-infrared spectroscopy study. PLoS One.

[50] Schneider W, Eschman A, Zuccolotto A (2012). E-Prime User’s Guide.

[51] Randolf C, Tierney MC, Mohr E, Chase TN (1998). The repeatable battery for the assessment of Neuro-psychological status (RBANS): preliminary clinical validity. J Clin Exp Neuropsychol.

[52] Guralnik JM, Simonsick EM, Ferrucci L (1994). A short physical performance battery assessing lower extremity function: association with self-reported disability and prediction of mortality and nursing home admission. J Gerontol.

[53] Motl RW, Dlugonski D, Suh Y (2010). Multiple Sclerosis Walking Scale-12 and oxygen cost of walking. Gait Posture.

[54] Motl RW, Weikert M, Suh Y (2012). Accuracy of the actibelt(®) accelerometer for measuring walking speed in a controlled environment among persons with multiple sclerosis. Gait Posture.

[55] Izzetoglu M, Chitrapu P, Bunce S, Onaral B (2010). Motion artifact cancellation in NIR spectroscopy using discrete Kalman filtering. Biomed Eng Online.

[56] Boas DA, Franceschini MA, Dunn AK, Strangman G. Noninvasive Imaging of Cerebral Activation with Diffuse Optical Tomography. In: Frostig RD, ed. In-vivo optical imaging of brain function. Boca Raton: CRC Press. 2002. 26844320

[57] R Development Core Team (2014). R: A Language and Environment for Statistical Computing.

[58] Bates D, Maechler M, Bolker BM, Walker S. Fitting Linear Mixed-Effects Models using lme4. J Stat Softw. 2015;67:1-48.

[59] Jenkins IH, Brooks DJ, Nixon PD, Frackowiak RS, Passingham RE (1994). Motor sequence learning: a study with positron emission tomography. J Neurosci.

[60] Jueptner M, Stephan KM, Frith CD, Brooks DJ, Frackowiak RS, Passingham RE (1997). Anatomy of learning. I. Frontal cortex and attention to action. J Neurophysiol.

[61] Jueptner M, Frith CD, Brooks DJ, Frackowiak RS, Passingham RE (1997). Anatomy of motor learning. II. Subcortical structures and learning by trial and error. J Neurophysiol.

[62] Passingham RE (1996). Attention to action. Philos Trans R Soc Lond Ser B Biol Sci.

[63] Toni I, Krams M, Turner R, Passingham RE (1998). The time course of changes during motor sequence learning: a whole-brain fMRI study. NeuroImage.

[64] Holtzer R, Mahoney JR, Izzetoglu M, Izzetoglu K, Onaral B, Verghese J (2011). fNIRS study of walking and walking while talking in young and old individuals. J Gerontol A Biol Sci Med Sci.

[65] Holtzer R, Mahoney JR, Izzetoglu M, Wang C, England S, Verghese J (2015). Online fronto-cortical control of simple and attention-demanding locomotion in humans. NeuroImage.

[66] Al-Yahya E, Johansen-Berg H, Kischka U, Zarei M, Cockburn J, Dawes H. Prefrontal cortex activation while walking under dual-task conditions in stroke: A multimodal imaging study. Neurorehabil Neural Repair. 2016;30(6):591-599. 10.1177/1545968315613864 PMC5404717 26493732

[67] Reuter-Lorenz PA, Cappell KA (2008). Neurocognitive aging and the compensation hypothesis. Curr Dir Psychol Sci.

[68] Rypma B, Berger JS, D'Esposito M (2002). The influence of working-memory demand and subject performance on prefrontal cortical activity. J Cogn Neurosci.

[69] Filippi M, Rocca MA, Colombo B, Falini A, Codella M, Scotti G, Comi G (2002). Functional magnetic resonance imaging correlates of fatigue in multiple sclerosis. NeuroImage.

[70] Lee M, Reddy H, Johansen-Berg H (2000). The motor cortex shows adaptive functional changes to brain injury from multiple sclerosis. Ann Neurol.

[71] Rocca MA, Falini A, Colombo B, Scotti G, Comi G, Filippi M (2002). Adaptive functional changes in the cerebral cortex of patients with nondisabling multiple sclerosis with the extent of brain structural damage. Ann Neurol.

[72] D’Esposito M, Onishi K, Tompson H, Robinson K, Armstrong C, Grossman M (1996). Working memory impairment in multiple sclerosis: evidence from a dual task paradigm. Neuropsychology.

[73] Harada T, Miyai I, Suzuki M, Kubota K. Gait capacity affects cortical activation patterns related to speed control in the elderly. Exp Brain Res. 2009;193(3):445–54. 10.1007/s00221-008-1643-y 19030850

[74] Kaoru Takakusaki. Forebrain control of locomotor behaviors. Brain Research Reviews. 2008;57(1):192-198. 10.1016/j.brainresrev.2007.06.024 17764749

[75] Holtzer R, Epstein N, Mahoney JR, Izzetoglu M, Blumen HM. Neuroimaging of Mobility in Aging: A Targeted Review. The Journals of Gerontology Series A: Biological Sciences and Medical Sciences. 2014;69(11):1375-1388. 10.1093/gerona/glu052 PMC4204614 24739495

[76] Yuan J, Blumen HM, Verghese J, Holtzer R. Functional connectivity associated with gait velocity during walking and walking-while-talking in aging: A resting-state fMRI study. Human Brain Mapping. 2015;36(4):1484-1493. 10.1002/hbm.22717 PMC4373975 25504964

